# Asymmetry of the Frontal Aslant Tract and Development of Supplementary Motor Area Syndrome

**DOI:** 10.3390/cancers16223739

**Published:** 2024-11-05

**Authors:** Jahard M. Aliaga-Arias, Josephine Jung, Jose Pedro Lavrador, Kapil Rajwani, Ana Mirallave-Pescador, Amy Jones, Hilary Wren, Richard Gullan, Ranj Bhangoo, Keyoumars Ashkan, Flavio Dell’Acqua, Francesco Vergani

**Affiliations:** 1 Department of Neurosurgery, King’s College Hospital NHS Foundation Trust, London SE5 9RS, UK; josephine.jung@nhs.net (J.J.); josepedro.lavrador@nhs.net (J.P.L.); k.rajwani@nhs.net (K.R.); richardgullan@nhs.net (R.G.); ranj.bhangoo@nhs.net (R.B.); k.ashkan@nhs.net (K.A.); 2 Institute of Psychiatry, Psychology & Neuroscience, King’s College London, London SE5 8AB, UK; 3 Department of Neurophysiology, King’s College Hospital NHS Foundation Trust, London SE5 9RS, UK; a.mirallave-pescador@nhs.net; 4 Department of Physiotherapy, King’s College Hospital NHS Foundation Trust, London SE5 9RS, UK; amy.jones29@nhs.net; 5 Department of Speech and Language Therapy, King’s College Hospital NHS Foundation Trust, London SE5 9RS, UK; hilary.wren@nhs.net; 6 Department of Basic & Clinical Neuroscience, King’s College London, London SE5 9RT, UK; 7 Natbrain Lab, Department of Forensic and Neurodevelopmental Sciences, King’s College London, London SE5 8AB, UK; flavio.dellacqua@kcl.ac.uk

**Keywords:** tractography, frontal aslant tract, supplementary motor area, low grade glioma, onco-functional neurosurgery

## Abstract

**Background/Objectives:** The purpose of this study was to investigate preoperative interhemispheric differences of the FAT in relation to the onset of postoperative SMA syndrome. **Methods:** This was a single-center retrospective analysis of patients who underwent surgical resection of diffuse gliomas involving the SMA between 2018 and 2022. Inclusion criteria were availability of preoperative and postoperative Magnetic Resonance Imaging, no previous surgery, and no neurological deficits at presentation. Diffusion-weighted data were processed by spherical deconvolution (SD) and diffusion tensor imaging tractography algorithms, and TrackVis was used to dissect the FAT of both hemispheres. The FAT data were analyzed for correlation with postoperative SMA syndrome onset. **Results:** *N* = 25 cases were included in the study, among which *n* = 23 had preoperative bilaterally identifiable FAT by SD. *N* = 12 developed an SMA syndrome, 6 demonstrated a motor-only syndrome, 4 had a verbal-only syndrome, and 2 had mixed verbal and motor features. The SMA syndrome incidence was significantly more frequent in lower-grade gliomas (*p* = 0.005). On the tumor side, the FAT identified by SD was smaller than the contralateral (mean volume 6.53 cm^3^ and 13.33 cm^3^, respectively, *p* < 0.001). In the 6 cases that developed a verbal SMA syndrome, a normalized FAT volume asymmetry (FAT-VA) demonstrated an asymmetry shifted towards the non-dominant side (mean FAT-VA = −0.68), while the cases with no postoperative verbal impairment had opposite asymmetry towards the dominant side (mean FAT-VA = 0.42, *p* = 0.010). **Conclusions:** Preoperative interhemispheric FAT volume asymmetry estimated according to functional dominance can predict postoperative onset of verbal SMA syndrome, with proportionally smaller FAT on the affected dominant hemisphere.

## Simple Summary

Mainstay endpoints of oncological neurosurgery include preservation of function and quality of life while achieving a maximal tumor resection. Surgical resection of tumors in the Supplementary Motor Area (SMA) can cause a syndrome characterized by impaired speech and movement initiation. The pathophysiological mechanisms are not clear yet, but increasing evidence points towards white matter pathways subserving this region, particularly the Frontal Aslant Tract (FAT), which connects the SMA to the inferior frontal gyrus. In this study, we have analyzed state-of-the-art spherical deconvolution tractography data from 25 consecutive patients to determine the influence of preoperative interhemispheric differences of the FAT on the development of the SMA syndrome. Proportionally smaller FAT volumes on the dominant hemisphere compared to the nondominant side were associated with an increased postoperative risk of verbal impairment (*p* = 0.010), demonstrating that preoperative FAT volume asymmetry estimated according to dominance can predict the onset of a verbal SMA syndrome.

## 1. Introduction

The frontal lobe is frequently involved in diffuse gliomas, and low-grade gliomas are more frequently located in the Supplementary Motor Area (SMA) within the frontal lobe in comparison to high-grade gliomas [1]. The SMA has been recognized to have a critical role in the higher-order control of movement and speech, being involved in motor initiation and coordination, as well as in spontaneous speech and verbal fluency [2,3,4,5]. The SMA can be divided into pre-SMA and SMA proper, separated by the vertical commissure anterior (VCA) line projection crossing the Superior Frontal Gyrus (SFG) [2]. A proposed functional organization model of the SMA describes the pre-SMA being connected to cognitively relevant cortical regions and the SMA proper being connected mainly to motor and premotor regions [4].

Surgery in this area and disconnection of related white matter tracts can lead to the post-operative SMA syndrome, which is characterized by movement reduction up to akinesia and speech impairment up to mutism, most frequently when the dominant hemisphere is involved [2,6,7,8]. The syndrome is frequently transient, but duration may range from one day to one year, and symptoms can rarely persist indefinitely [7,9]. The impact that the SMA syndrome can have on the onco-functional outcome emphasizes the necessity to investigate the mechanisms of development of the syndrome [10].

The Frontal Aslant Tract (FAT) has been identified as a tract that plays an important role in defining the SMA connectivity. The FAT connects the SMA (both pre-SMA and SMA-proper) laterally with the inferior frontal gyrus, mainly the *pars opercularis*. The FAT on the dominant side is involved in language (verbal fluency, initiation and inhibition of speech, sentence production, and lexical decision) [4,5,11,12]. The FAT has also been implicated in working memory, visual-motor activities, orofacial movements, social communication tasks, attention, and music processing [3,4,5,11,13,14,15].

Published literature suggested that the involvement or damage of the FAT in gliomas can explain the development of a postoperative SMA syndrome [11]. However, previous studies investigating the relevance of fiber tracts in the development of the SMA syndrome have produced mixed results and have been mainly conducted using the diffusion tensor imaging (DTI) technique [2,7,11,16,17]. In comparison, spherical deconvolution (SD) tractography has the advantage of modeling fiber tracts crossing in the same voxels and can provide along-tract measurements that correlate with the microstructural integrity of a specific fiber tract [18,19].

In this study, we investigate the role of the interhemispheric differences of the FAT in the development of postoperative SMA syndrome using SD and DTI tractography in patients undergoing resection of brain tumors located in the SMA region in patients with no previous neurological deficit. This information can be useful in estimating the risk of onset of postoperative SMA syndrome (crucial in counseling patients) and providing guidance for surgical strategy and perioperative clinical management.

## 2. Materials and Methods

### 2.1. Study Design

This was a single-center, retrospective cohort study of patients undergoing surgical resection for a diffuse glioma involving the SMA between 2018 and 2022 in a tertiary neurosurgical center. Inclusion criteria were availability of past medical history, pre- and postoperative clinical assessments, advanced Magnetic Resonance Imaging (MRI) with diffusion sequences, postoperative physiotherapy, and/or occupational therapy assessments. Patients with history of previous surgical resection of a diffuse glioma and/or with preoperative motor or speech deficits were excluded.

### 2.2. Data Collection and Outcome Measures

Anonymized data were collected into a secure database by accessing the electronic patient records (EPR). Functional outcomes were recorded from physiotherapy and occupational therapy assessments within the first 48 h after surgery (reporting the onset of focal neurological deficits and motor strength of the four limbs according to the Medical Research Council (MRC) score and functional assessments). Language deficits were collected from speech and language therapist reviews, including characterization of spontaneous speech and the Sheffield Screening Test for Acquired Language Disorders (SST), categorizing dysphasia into receptive and expressive components [20].

The variables collected included demographics, handedness, medical comorbidities, preoperative focal neurological deficits, surgical protocol (awake vs. asleep), WHO CNS tumor classification, postoperative MRC and motor control deficits, postoperative speech deficits, length of stay, discharge destination, time to onset of SMA syndrome, and if present at discharge. In accordance with previous literature, the occurrence of acute postoperative motor SMA syndrome was categorized by the presence of motor deficits, which included a combination of motor initiation, bimanual task and dexterity impairments, or akinesia of one limb. The verbal SMA syndrome was defined by the presence of a language dysfunction detected by the SST battery and mainly characterized by spontaneous speech deficit (delayed initiation and reduced length of speech) or by pure mutism with preserved comprehension [2,5,11]. Subcategories of pure motor, pure verbal, or mixed syndromes were also clustered. Severe SMA syndrome cases were considered if MRC was <3 in at least one limb or if severe dysphasia was reported (corresponding to a score of <8 combined on the SST).

### 2.3. Imaging and Tractography Data

MRI data has been acquired from a 1.5 Tesla Siemens Magnetom Aera scanner (Siemens AG, Erlangen, Germany) using a standard 20-channel head/neck receive coil. Diffusion data were acquired using a single-shot spin echo EPI sequence with the following parameters: TE = 86 ms, TR = 9500 ms, 48 axial slices, slice thickness = 2.5 mm, acquisition matrix 128 × 128 with a field of view of 320 × 320 mm and isotropic spatial resolution of 2.5 × 2.5 × 2.5 mm, parallel imaging acceleration GRAPPA = 2. Diffusion-weighting imaging data (DWI) were acquired with a b-value of 1500 s/mm^2^ along 64 gradient directions and 6 additional non-diffusion-weighted (b = 0) volumes. The radiological data also included a preoperative MRI with T1 volumetric post-gadolinium and either volumetric T2 or FLAIR. Radiological features reported included anatomical description of tumor location, preoperative tumor volume, and involvement of SMA. Preoperative tumor volumes were measured by segmentation on Stealth S8 software, based on T1 post-gadolinium (high-grade gliomas) or T2/FLAIR sequences (low-grade gliomas). A post-operative MRI performed within 72 h (high-grade gliomas) or at 1 to 3 months (low-grade gliomas) after surgical intervention was used to determine the post-operative tumor residue volumes (calculated as above). The percentual volumetric extent of resection (EoR) was also estimated [21].

Diffusion data were first denoised and corrected for Gibbs ringing, motion, and eddy current distortion [22,23,24]. StarTrack software Version 2022-01-27 was then used to perform DTI and SD tractography (https://www.mr-startrack.com/, accessed on 1 February 2023). SD was performed using the dRL algorithm (fiber response parameter ALFA = 1.5, 400 iterations, regularization η = 0.0015, ν = 16). Deterministic tracking was performed with an angle threshold of 45°, an absolute fODF amplitude threshold of 0.0025, and a step size of 1 mm. For comparison, DTI tractography was also performed with nonlinear least squares diffusion tensor fitting. Deterministic tractography was performed using a Euler interpolation with an FA threshold of 0.15, a 35-degree angle threshold, and a step size of 1 mm tracking. The trajectory streamlines included had minimum length of 20 mm and maximum length of 300 mm.

The digital dissection of the tracts was performed using Trackvis (trackvis.org, accessed on 1 May 2023) with a two region of interest (ROI) technique, outlined freehand on 2D multiplanar projections in the three planes (axial, coronal, and sagittal) from the volumetric sequences available from the acquisitions described above. The ROIs were designed according to previous anatomical and tractography literature [3,13,25,26]. Exclusion ROIs were created based on prior anatomical knowledge to eliminate spurious streamlines. To identify the FAT, for each hemisphere, one ROI was located on the superior frontal gyrus at the level of the SMA and the other covering the posterior portion of the IFG (at the pars opercularis/triangularis) and anterior-inferior portion of the precentral gyrus. The virtual dissections were performed by one investigator (JMAA) on anonymized datasets and blinded to the clinical outcomes and reviewed by two senior authors (FDA and FV). The variables of the tracts estimated and recorded were volume, mean length, mean apparent diffusion coefficient (ADC), and mean hindrance-modulated orientation anisotropy (HMOA) available only for the SD data [18,27].

### 2.4. Data Processing and Statistical Analysis

Statistical analysis was performed using IBM® SPSS® Statistics version 29. Preoperative patient demographic and clinical features, surgical protocol, and pathological features were analyzed for possible association with postoperative SMA syndrome onset and clinical course. Tractography variables were compared between hemispheres ipsilateral and contralateral to the tumor side. Interhemispheric differences in the tract values were analyzed for association with SMA syndrome. Interhemispheric differences were additionally evaluated. The tracts from the affected sides were considered evaluable as the cases selected had no preoperative motor nor language alterations.

A normalized index of interhemispheric tract volume asymmetry (VA) was calculated by estimating the interhemispheric difference between the volumes of the tracts divided by their average, as previously reported in the literature [3]. The VA was calculated according to the dominant side, defined by the following Formula (1):

VA = (dominant side tract volume − nondominant side tract volume)/[(dominant side tract volume + nondominant side tract volume)/2].(1)

Positive VA indicated a proportionally larger tract volume on the dominant side, and a negative index indicated a smaller tract volume on the dominant side.

The Shapiro–Wilk test was performed for determination of normality of variables; thereafter, for parametric variables, the *t*-test was used for comparison of means, ANOVA, and Pearson test for correlation; for non-parametric variables, Levene’s test was used for equality of variances, Mann–Whitney U test, and Spearman were used for comparison and correlation of medians, respectively. Categorical associations were evaluated by Pearson Chi-square or Fisher tests for comparison of preoperative factors among case groups and odds ratios for exposures to determinants of outcome such as tumor location and anesthetic protocol. Somers’ D was used for ordinal nonparametric associations. Statistical significance was attributed to *p* values below 0.05.

## 3. Results

### 3.1. Sample Size and Exclusion Report

Overall, 33 cases were identified to have undergone surgical resection of diffuse glioma involving the SMA region. Three cases were excluded due to having had previous surgery; four cases were excluded due to preoperative motor deficits (*n* = 2) and dysphasia (*n* = 2). One case was excluded as no diffusion-weighted imaging sequences had been acquired to be processed for tractography. Therefore, 25 cases were included in the study analysis.

### 3.2. Patient Variables and SMA Syndrome Incidence

The mean age of the 25 patients was 44 years (range 22–71 years). The male-to-female ratio was 0.92. Twelve cases had an acute postoperative SMA syndrome (46%) (Table 1). No preoperative patient-specific variable showed a statistically significant difference concerning the development of the SMA syndrome (Table 1). Among the 12 affected cases, four patients (33%) developed the SMA syndrome within a few hours after surgery, while the other eight patients (67%) showed deterioration on the first postoperative day.

### 3.3. Anatomical, Surgical, and Pathological Variables

A total of 17 tumors (68%) were located in the nondominant hemisphere; 18 tumors (72%) involved the SMA proper, 22 involved the pre-SMA (88%), and 15 involved the whole SMA (60%). The median preoperative tumor volume was 46.8 cm^3^ (range 3.6–195.9 cm^3^). No significant differences in anatomical location and volumetric tumor variables were related to the postoperative development of SMA syndrome (Table 1). Among 18 patients (72%) who underwent awake surgery, 11 had a tumor on the nondominant side (61%). The median resection volume was 43.8 cm^3^ (range 3.6–195.9 cm^3^) and the median EoR was 96.51% (range 29.68–100%). Patient features, surgical protocol, and EoR had no significant associations with the development of SMA syndrome (Table 1 and Table S1).

**Figure 1:**
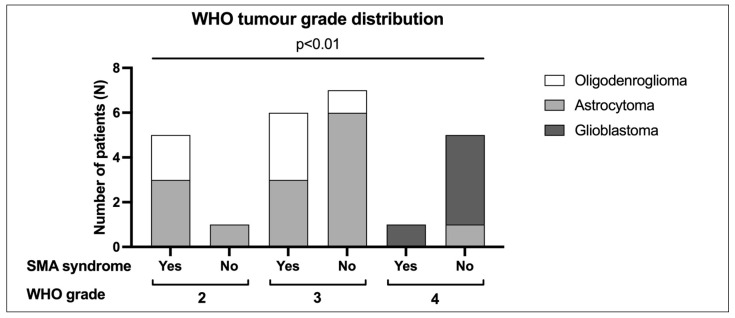
Distribution of WHO grading among subgroups of patients with or without SMA syndrome onset. N = 6 were IDH-mutant WHO grade 2 gliomas (n = 2 oligodendrogliomas, n = 4 diffuse astrocytomas), n = 13 IDH-mutant WHO grade 3 gliomas (n = 4 oligodendrogliomas 1p/19q-codeleted, n = 9 diffuse astrocytomas), n = 9 WHO grade 4 gliomas (n = 1 IDH-mutant astrocytoma, grade 4; n = 5 glioblastomas, IDH wild-type). The SMA syndrome occurrence was more frequent in lower WHO grade tumors when compared with WHO grade 4 tumors (5/6 WHO grade 2, 6/13 in WHO grade 3, and 1/6 WHO grade 4, Somers’ D value −0.437, *p* = 0.005).

In total, there were 6 IDH-mutant WHO grade 2 diffuse gliomas (2 oligodendrogliomas, 4 astrocytomas), 13 IDH-mutant WHO grade 3 diffuse gliomas (4 oligodendrogliomas, 9 astrocytomas) and 6 WHO grade 4 diffuse gliomas (1 IDH-mutant astrocytoma, 5 glioblastomas). The SMA syndrome occurrence was more frequent in lower WHO grade tumors when compared with WHO grade 4 tumors (5/6 WHO grade 2, 6/13 in WHO grade 3, and 1/6 WHO grade 4, Somers’ D value −0.437, *p* = 0.005) (Figure 1).

### 3.4. Functional Outcomes

Six cases demonstrated a motor-only syndrome, four a verbal-only syndrome, and two had a syndrome with mixed verbal and motor features (see Tables S2 and S3 for details). All patients with SMA syndrome underwent acute postoperative rehabilitation and occupational therapy. Among the cases affected by the syndrome, 11 showed improvement of the condition within a week (92%), but only two cases had resolution of the syndrome within a week (17%). As expected, the median postoperative length of stay (LoS) was significantly longer in the patients that developed the SMA syndrome (6.5 days, IR 4 days) with respect to those that did not (3 days, IR 3 days, *p* = 0.007). Among the other variables considered (age, tumor size and grading, extent of resection, type of surgery), only age correlated directly with the length of stay (r = 0.462, *p* = 0.020), with older patients tending to have a longer stay. Five patients required transfer to a rehabilitation unit (20%); all had resection of tumors involving globally both the pre-SMA and the SMA proper, and four of them had a motor SMA syndrome.

### 3.5. Interhemispheric Differences of FAT

**Figure 2:**
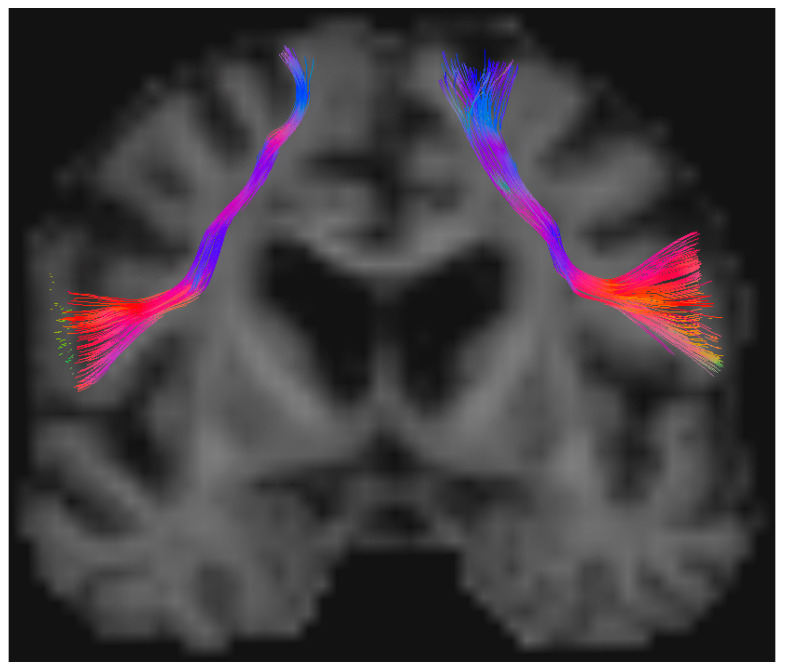
Example of spherical deconvolution tractography dissection of FAT in both hemispheres performed with TrackVis (trackvis.org, accessed on 1 May 2023), with bilaterally identifiable tract. Regions of interest are located in SMA on the superior frontal gyrus and posterior inferior frontal gyrus. Fibers projecting from the SMA to the anterior subcentral gyrus, pars opercularis, and posterior pars triangularis. In this case, the tumor is located in the right hemisphere.

The SD tractography dissection of the FAT (Figure 2) demonstrated bilaterally identifiable tracts in 23 cases (92%), and among these, all the FAT variables estimated (i.e., volume, mean length, mean HMOA, mean ADC) were statistically different between hemispheres homolateral and contralateral to tumor side.

As expected, the FAT on the tumor side had smaller volumes (means 6.53 mm^3^ vs. 13.33 mm^3^, *p* < 0.001) and was longer (means 74.24 mm vs. 69.90 mm, *p* = 0.043) in comparison to the contralateral tract when identified with SD. With this technique, the tumor-side mean HMOA values of the FAT were significantly lower (HMOA 0.011 vs. 0.013, *p* = 0.001) and the mean ADC significantly higher (0.770 × 10^−3^ mm^2^/s vs. 0.682 × 10^−3^ mm^2^/s, *p* < 0.001) in comparison to the contralateral side. These changes are likely to reflect the impact of the tumor on the affected side (Table 2).

The interhemispheric FAT volume asymmetry (VA) estimated with SD data were compared for the cases with lesions on the dominant side and those with tumors in the nondominant hemisphere. Concordantly with the effect demonstrated on the single hemisphere, the VA was oriented contralaterally to the lesions, with negative values for cases with tumors on the dominant side (mean −0.92) and positive values in those with tumors in the nondominant hemisphere (mean 0.70, *p* < 0.001) (Figure S1).

The DTI tractography dissection of the FAT demonstrated bilaterally identifiable tracts in 17 cases (68%). In these cases, the DTI data detected longer FATs on the tumor side (means 72.34 mm vs. 68.10 mm, *p* = 0.036) and increased ADC (0.744 × 10^−3^ mm^2^/s vs. 0.682 × 10^−3^ mm^2^/s, *p* = 0.003) compared to the tracts contralateral to the lesions, showing tendencies similar to those detected by SD tractography. The FAT volumes estimated by the DTI did not demonstrate significant differences between those on the tumor side compared to the contralateral side (respectively means 5.54 mm^3^ vs. 7.79 mm^3^, *p* = 0.053), though showing a tendency for a smaller tract on the tumor side as seen with the SD technique.

Given its reduced consistency in identifying the FAT and the limited sensitivity in detecting differences in volume induced by the tumor, the data from the DTI tractography were not considered relayable enough for the estimation of the interhemispheric asymmetry and for the functional correlations of the tract variables with the SMA syndrome onset. Therefore, the following results referring to the estimates of interhemispheric differences of the FAT were obtained only from the SD tractography data.

### 3.6. Verbal SMA Syndrome and Interhemispheric FAT Asymmetry

**Figure 3:**
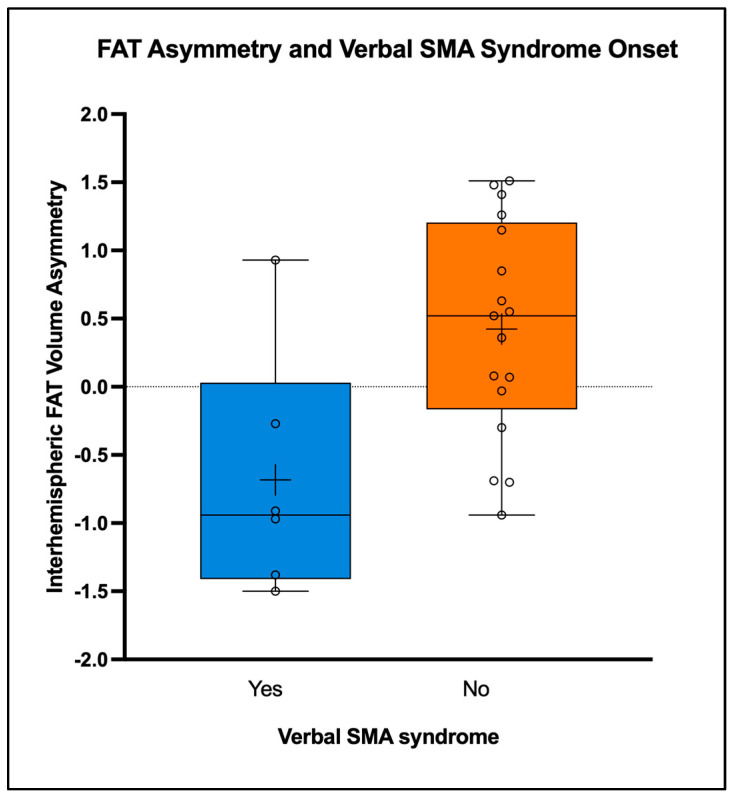
Box plot representations of mean values of spherical deconvolution interhemispheric FAT volume asymmetry (VA) estimated according to dominance, plotted for the verbal SMA syndrome onset and no verbal syndrome groups (respectively mean VAs −0.68 and 0.42, *p* = 0.010). The cases affected by the verbal syndrome have a negative mean VA value, therefore asymmetry oriented towards the nondominant hemisphere, while the non-affected cases have a positive mean VA oriented towards the dominant hemisphere.

Among the 23 cases with bilaterally identifiable FAT with SD tractography, 11 developed an SMA syndrome (48%). Given the predominant role of FAT in language, we performed an analysis of FAT asymmetry in relationship with the onset of verbal SMA syndrome [5,11]. The comparison of the interhemispheric ratio of FAT volumes showed significant differences. In the six cases that developed a postoperative verbal SMA syndrome, the FAT volumes on the dominant side were proportionally smaller than the FAT on the non-dominant side, with normalized asymmetry (VA) values showing significant differences associated with the verbal outcomes. The six cases that developed a verbal SMA syndrome had a VA oriented towards the non-dominant side (mean VA = −0.68, SD = 0.904), while the 17 cases that had no verbal compromise had an oppositely oriented asymmetry (mean VA = 0.42, SD = 0.793, *p* = 0.010) (Table 3 and Figure 3).

### 3.7. FAT Variables Associated with SMA Syndrome Onset

The tractography-derived variables determined by SD tractography (mean volume, mean length, mean HMOA, and mean ADC) of the FATs on the tumor side (Table S4) and of the FATs on the contralateral side (Table S5) were not statistically different between patients that developed an SMA syndrome and those that did not. The FAT volumes on the dominant side were not significantly different between the cases that developed the verbal SMA syndrome and those that did not have this impairment after surgery (respectively means 7.25 mm^3^ and 11.47 mm^3^, *p* = 0.13). In two cases, the FAT was not preoperatively identifiable by SD on the tumor side, but only on the contralateral side. One of these two patients developed an SMA syndrome.

The VA was not associated with the development of the motor syndrome or SMA syndrome considered in general, as reported in Tables S6 and S7. When tested in the subgroups of SMA syndrome and non-SMA syndrome, the VA maintained the orientation concordant to the lesion laterality in both subgroups, negative when the lesion was on the dominant side and positive when in the nondominant hemisphere, as reported in the Supplementary Figure S2.

The DTI tractography could not identify the FAT on the tumor side but only the contralateral side in seven cases, of which four developed an SMA syndrome. In one case, the DTI could not demonstrate the FAT on the side contralateral to the tumor but could depict it on the side of the tumor, which did not develop the SMA syndrome. The inconsistency of the DTI results prevented further evaluation of associations of the tract variables with the clinical outcomes.

### 3.8. Illustrative Case

**Figure 4:**
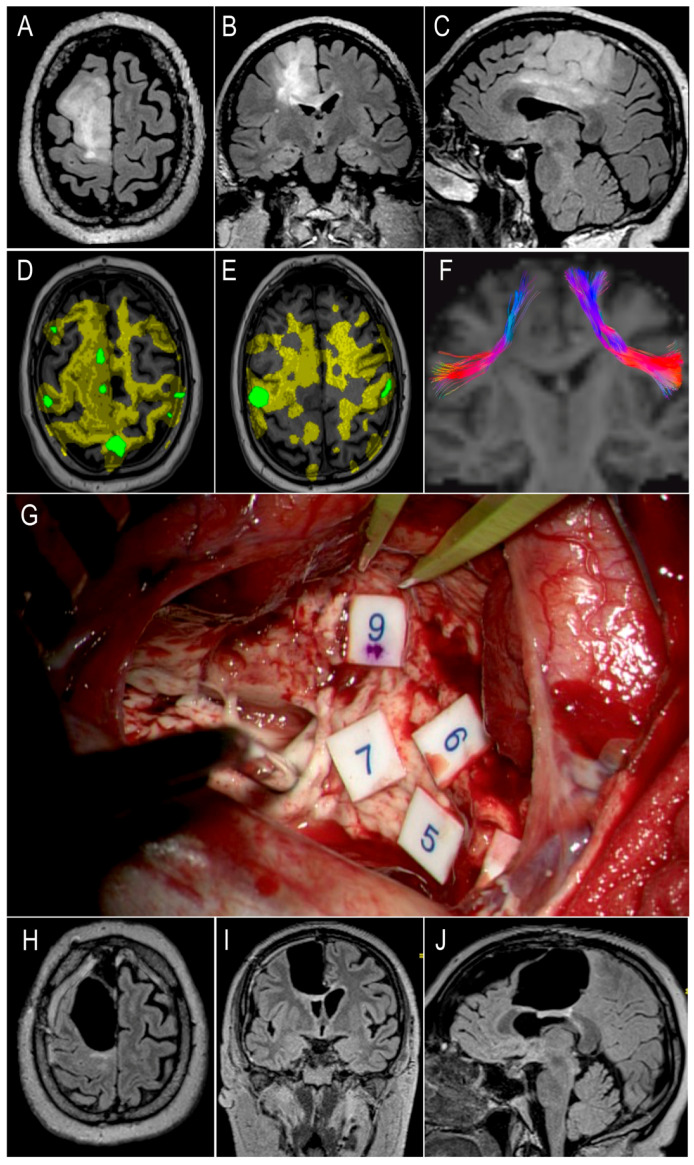
Illustrative case: 55-year-old patient who developed postoperative verbal SMA syndrome. Panels: (**A**–**C**) preoperative MRI demonstrating a hyperintense lesion in the SMA extending into the corpus callosum on FLAIR sequences in axial, coronal and sagittal planes respectively; (**D**) fMRI reconstructions of activations detected for verbal production and (**E**) verbal fluency testing with color maps for different activation thresholds (yellow for >30% of the maximum activation, green for >60% of the maximum activation), demonstrating right lateralization of language and activations at the level of the right SMA for verb generation; (**F**) SD tractography of bilateral FAT, showing smaller tract on the right dominant hemisphere; (**G**) resection cavity demonstrating access to lateral ventricle with choroid plexus exposed and skeletonized CST mapped with high frequency monopolar DES (tags 5, 6 and 7 marking motor upper limb responses at 5 mA, tag 9 marking motor upper limb and face responses at 5 mA); (**H**–**J**) postoperative MRI demonstrating resection cavity at the level of the right SMA extending to the corpus callosum and lateral ventricle on FLAIR sequences in axial, coronal, sagittal planes respectively.

A 55-year-old, left-handed patient presented with generalized, tonic-clonic seizures (case n 4 in Tables S1 and S2). The preoperative MRI demonstrated a non-enhancing lesion of the right SMA extending to the corpus callosum (Figure 4, panels A–C). The preoperative SLT evaluation was within normal limits (SST score of 19/20, 11 expressive, and 8 receptive). The preoperative fMRI demonstrated a right-sided lateralization of speech (Figure 4, panels D and E). The patient underwent an awake craniotomy with direct electrical stimulation (DES) mapping with language and motor tasks, achieving a subtotal resection (EoR 95%) extended up to anatomical and functional limits, which at the posterior margin were defined by motor responses elicited from the cortico-spinal tract (Figure 4, panels G–L). Toward the end of the resection, the patient developed episodes of spontaneous speech arrest, initiation disturbance, and hesitation, not reproducible with DES. Immediately after surgery, the patient developed a mixed motor and verbal SMA syndrome. The patient received physiotherapy and showed improvement in strength on reassessment 7 days after surgery but required transfer to a rehabilitation unit. SD tractography demonstrated that the dominant FAT was 5.92 cm^3^, smaller compared to the nondominant FAT (15.77 cm^3^) (Figure 4, panel F). The estimated FAT-VA was −0.91, corresponding to the development of verbal SMA syndrome in this case. The diagnosis was IDH-mutant, 1p/19q-codeleted oligodendroglioma WHO grade 3.

## 4. Discussion

The SMA syndrome was first described in a series of three patients by Laplane in 1977 after resection of the dorso-medial prefrontal lobe. Remarkably, all the patients had a postoperative speech impairment, but two had a right-sided resection, and all were right-handed [8]. Due to its variability in presentation and despite multiple studies investigating the functions of the SMA and its related connectivity, a conclusive answer regarding the causative mechanism of the syndrome remains elusive [6,16].

We report a consecutive series of 25 patients who underwent resection of a diffuse glioma involving the SMA region. Our sample was well balanced regarding patient demographic features and representative of the neuro-oncological population affected by SMA tumors [28,29]. In our series, 46% of patients developed an SMA syndrome, which is also in line with previous reports from the literature, with the incidence of the syndrome ranging from 23% to 100% [2]. SMA syndrome was significantly more frequent in lower-grade gliomas. Overall, FAT-derived metrics were not related to the postoperative development of SMA syndrome. However, FAT interhemispheric asymmetry was significantly related to verbal SMA syndrome after tumor resection—normalized values according to dominance oriented towards the non-dominant hemisphere.

Data from the literature have shown that the onset of SMA syndrome is possibly associated with the extent of resection of the SMA region. Previous studies suggested that resection of the medial and posterior wall of the SMA [30], or an extent of resection superior to 90% of the SMA region, is associated with a higher incidence of SMA syndrome after surgery [31]. An increased risk of SMA syndrome has been described when trespassing the medial part of the SMA and the adjacent cingulate gyrus [2]. This may be due to interruption of the nearby callosal association fibers, as the contralateral SMA has a particular important function in brain plasticity, and damage to this medial component and the adjacent middle cingulate gyrus seems to be related also to a longer length of recovery. In the present study, patients who developed an SMA syndrome tended to have larger tumors (median preop volume 66.15 cm^3^ vs. 46.10 cm^3^) and larger resections (median resection volume 50.25 cm^3^ vs. 43.80 cm^3^), although this difference did not reach statistical significance. This may be related to the relatively small sample of patients in the present cohort. Similarly, no association was found between the development of the syndrome and anatomical extension of the tumor (e.g., global SMA involvement, cingulate invasion, etc.) or surgical protocol employed (awake or asleep surgery). With regard to the pathological feature of gliomas, in our series WHO grade 2 was correlated to a higher risk of developing an SMA syndrome, as opposed to WHO grades 3 and 4. This is in line with previous reports from the literature and is likely to reflect the infiltrative nature of WHO grade 2 gliomas that can extend into functional tissue (with occasional positive functional responses observed inside the tumor) [31,32].

In recent years, there has been a growing interest in the role played by white matter connections in the genesis of the SMA syndrome. The results reported in the literature, however, have not been conclusive. Kinoshita et al. analyzed postoperative tractography and resection cavities, denoting that proximity of resections to the left FAT was associated with postoperative speech initiation dysfunction while proximity to either FST was associated with motor initiation disorder development [11]. However, neither the EoR nor the postoperative tract volumes were associated with the onset of an SMA syndrome [11]. Young et al. used DTI to depict the FAT before and after resection of tumors involving the SMA [16]. All patients with a preoperatively detectable tract and subsequent surgical disruption developed the SMA syndrome, but preservation of the FAT did not always prevent the SMA syndrome occurrence, and patients who had preoperative FAT disruption by the tumor could still develop the SMA syndrome postoperatively, concluding that the relationship between FAT and SMA syndrome is complex to define [16]. However, those findings suggest that a tract can be just masked by a tumor and remain functional even if not visible by tractography.

In our study, two out of 25 cases had no identifiable FAT on the tumor side employing SD tractography, and in these cases, the lack of detection of the tract could have been caused by either low b-value or data quality. The b-value of 1500 used in the acquisition of the diffusion sequences reflects the restraints of the clinical setting on which this study is based, with its limitations to the time- and cost-effectiveness. Although optimal b-values for SD have been reported to be 2000 or 3000, the use of b-1500 offers a compromise in terms of signal-to-noise ratio while still being able to resolve major crossing of fibers. Protocols with a higher b-value allow a higher chance of identification of the tract and better delineation of its structure but require longer acquisition times. Currently, the protocol does not permit free water elimination; however, future studies with an updated acquisition protocol could incorporate multi-shell acquisitions. This adjustment would enable free water elimination. Additionally, using high b-values already allows for the suppression of fast diffusion components, which may aid in reducing signal contributions from edema. The data currently reported adhere to the realistic oncological neurosurgery scenario in which these cases are commonly managed. As expected, using the same DWI data, the tractography performed with the DTI algorithm was able to identify the FAT in an inferior number of cases compared to the SD algorithm, accounting for only 18 out of 25 on the tumor side. This result confirms a general superiority of the SD in delineating tracts in complex anatomical locations compared to single tensor tractography, also in the clinical setting [18,19,27].

In our study, the SD algorithm demonstrated a FAT on the tumor side had significantly smaller volumes and increased length in comparison to the contralateral tract. Tumor-side HMOA values of the FAT were significantly lower and the ADC significantly higher. These findings likely reflect the distortion and infiltration or compression of the tract by the tumor. The DTI analysis showed similar results for FAT length and ADC (respectively longer and higher in FAT on the tumor side compared to the contralateral), but did not demonstrate a significant difference of the FAT volumes among hemispheres affected by tumor and the contralateral hemisphere. These results demonstrated a limitation of the DTI in identifying structural differences of the FAT when involved by a tumor and prevented the possibility to employ this technique for estimation of the tract volume asymmetry and the assessment of associations with functional outcomes. The worse performance of DTI can be attributable to the increased susceptibility of DTI to partial volume, affecting not only estimates of anisotropy but also fiber orientation for tractography.

The analysis on the SD data of FAT asymmetry according to, using the normalized VA indices, demonstrated that a smaller FAT in the dominant hemisphere (compared to the contralateral FAT) was associated with an increased risk of developing a verbal SMA syndrome. Our results are in line with the known role of FAT in speech production, given its connection with the pars opercularis of the inferior frontal gyrus, and specifically suggest that when the FAT is compromised on the dominant side, patients are at higher risk of having a speech deficit in the immediate postoperative period [4,5]. Of note, in our series, a verbal SMA syndrome was present also in a right-handed patient presenting with a right-sided tumor, in accordance with the original description of the syndrome by Laplane [8].

The unilateral volume of the dominant FAT was not associated with the development of the verbal SMA syndrome, therefore excluding the possibility of reducing the findings from the asymmetry analysis to the simple effect of the tumor. Previous anatomical data from healthy subjects have shown that the FAT tends to be larger on the dominant side, a fact that has been interpreted as further underpinning its role in language [3,13]. The fact that the “normal” asymmetry of the FAT (i.e., a larger tract on the dominant side) is reversed in the case of a verbal SMA syndrome can help to better understand the genesis of the syndrome itself. In this scenario, the verbal symptoms observed after surgery could originate from a functional imbalance between the two hemispheres driven by changes in the SMA connectivity, in a hodological interpretation of the SMA syndrome [17,33].

The asymmetry of the FAT was not associated with the onset of motor symptoms. This is perhaps not surprising, as an involvement of the FAT in the initiation and planning of the movement has not been fully demonstrated [11,14,34]. Other connections could help to better understand the role of the SMA in movement, such as short U-fiber connections between SMA-proper and primary motor cortex or direct connections to the striatum through the FST [3]. The asymmetry was concordant with the location of the tumor in the dominant or nondominant hemisphere, and this feature was maintained also when assessing the subgroups of cases that developed the SMA syndrome and cases with no postoperative syndrome.

The present study is somewhat limited by the relatively small sample of patients, particularly when considering the observed cases of SMA syndrome. Although our sample is comparable to previous reports from the literature, it is possible that some of the negative correlations observed (e.g., the relation between EoR and onset of SMA symptoms as discussed above) are due to the limited power of our analysis [7,16]. We acknowledge that the reduced sample size elicits caution when extending the results to the general neuro-oncological population. Having been able to identify significant associations with the functional outcomes and regarding the verbal SMA syndrome, this study nevertheless warrants further investigations with a larger sample and possibly multi-centric design to further validate the current results. In addition, the more relevant results observed have been obtained with specific parameters of DWI, namely using a Spherical Deconvolution algorithm. It remains to be demonstrated if they can be replicated by other centers, particularly when using different DWI protocols, which would be relevant to their clinical generalizability. SD has the advantage of differentiating between different directions in the same voxel, thus offering a solution to the problem of “crossing” fibers [18]. This is clearly useful in studying the white matter tracts of the SMA, where multiple fibers can cross and overlap (e.g., FAT and callosal radiations), thus making their reconstruction challenging with conventional DTI, as evidenced above. The SD protocols applied in this study have been proven to be reliable in a previous study from our group, correlating the presence of altered excitability of the motor cortex with microstructural changes of the cortico-spinal tract in patients with glioma [19]. In addition, we employed a previously validated normalization methodology to account for the effect that pathological findings (such as the presence of a tumor or a stroke) can have on the tract volumes [13,35]. Together with the inclusion selectivity for preoperatively functionally intact patients, these parameters allow reliability of the findings.

The results provided by this study demonstrate for the first time and to the best of the authors’ knowledge, that proportional interhemispheric changes to the structure of the FAT demonstrated by SD tractography have functional implications in surgery for the SMA. This represents, in our opinion, an innovation in the interpretation of the verbal SMA syndrome, stemming from previous literature pointing towards interhemispheric mechanisms underlying the development of this condition and to the role of the FAT in language. We consider the findings reported to encourage future research investing the role of the FAT asymmetry in the neuro-oncological setting also for other cognitive functions hypothesized to be supported by this tract [2,3,10,14]. Our results have a direct clinical relevance, particularly in the role of patient counseling ahead of surgery for low-grade gliomas involving the SMA region. The combination of structural MRI (with a clinical suspicion of a WHO grade 2 glioma) and diffusion-weighted imaging (showing a “reversed” asymmetry of the FAT, with a smaller tract on the dominant side) could help to inform patients about the risk of developing a verbal SMA syndrome. This has clear implications, with a prolonged in-hospital stay (as demonstrated in our study), need for postoperative rehabilitation, and time of recovery after surgery [2,9,16]. An informative patient counseling is essential in the definition of an individualized onco-functional plan, which can potentially modify the surgical planning according to the patient’s needs, for example, choosing to perform an awake surgery with intraoperative language mapping and monitoring. In addition, if a patient is recognized as at increased risk of developing a verbal syndrome, postoperative SLT involvement and acute rehabilitation can be planned in advance to increase efficiency of care and enhance recovery [11,12,33].

## 5. Conclusions

The SMA syndrome is frequently observed after tumor resection in the SMA region. It is observed more frequently in low-grade gliomas, and it is associated with increased LOS. Diffusion-derived metrics are not associated with global SMA syndrome. However, preoperative interhemispheric FAT volume ratio (lower in tumor-hemisphere) and estimated according to functional dominance (shift towards non-dominant hemisphere) can predict the risk of postoperative onset of verbal SMA syndrome.

## Figures and Tables

**Table 1:** Synthesis of clinical, pathological, and radiologic associations with Supplementary Motor Area (SMA) syndrome onset in the cohort (n = 25) and statistical tests employed and significance values. EoR—extent of resection; IR—interquartile range; LOS—length of stay; SD—standard deviation.

Variable			SMA Syndrome (N = 12)	No Syndrome (N = 13)	Total (N = 25)	Statistics
Mean Age (SD)			45 yrs (14 yrs)	43 yrs (15 yrs)	44 yrs (14 yrs)	*p* = 0.682
Gender	Female		7 (58%)	6 (46%)	13 (52%)	*p* = 0.695
	Male		5 (42%)	7 (54%)	12 (48%)	
Handedness	Left		1 (8%)	1 (8%)	2 (8%)	*p* = 1.000
	Right		11 (92%)	12 (92%)	23 (92%)	
Tumor Side	Dominant		6 (50%)	2 (15%)	8 (32%)	*p* = 0.097
	Nondominant		6 (50%)	11 (85%)	17 (68%)	
SMA-Proper Involvement			9 (75%)	9 (75%)	18 (72%)	*p* = 1.000
Pre-SMA Involvement			11 (92%)	11 (92%)	22 (88%)	*p* = 1.000
Global SMA Involvement			8 (67%)	8 (67%)	15 (60%)	*p* = 0.688
Cingulate Involvement			9 (75%)	9 (75%)	19 (76%)	*p* = 1.000
Awake Craniotomy			9 (75%)	9 (75%)	18 (72%)	OR 1.33 95% CI 0.23–7.74
Median Preoperative Volume (IR)			66.15 cm^3^ (89.3 cm^3^)	46.10 cm^3^ (27.90 cm^3^)	46.80 cm^3^ (54.65 cm^3^)	*p* = 0.320
Median Residue Volume (IR)			1.30 cm^3^ (8.8 cm^3^)	0.70 cm^3^ (4.25 cm^3^)	0.70 cm^3^ (6.35 cm^3^)	*p* = 0.852
Median Resection Volume (IR)			50.25 cm^3^ (74.82 cm^3^)	43.80 cm^3^ (25.40 cm^3^)	43.80 cm^3^ (44.95 cm^3^)	*p* = 0.503
Median Eor (IR)			97.27% (9.66%)	96.51% (9.60%)	96.51% (8.70%)	*p* = 0.936
Median Postoperative LOS (IR)			6.5 days (4 days)	3 days (3 days)	5 days (5 days)	***p*** **= 0.007**
IDH-Mut, 1p/19q Codel Oligodendroglioma		Grade 2	2 (17%)	0 (0%)	2 (8%)	***p*** **= 0.005**
		Grade 3	3 (25%)	1 (8%)	4 (16%)	
IDH-Mut Astrocytoma		Grade 2	3 (25%)	1 (8%)	4 (16%)	
		Grade 3	3 (25%)	6 (46%)	9 (36%)	
		Grade 4	0 (0%)	1 (8%)	1 (4%)	
IDH-Wt Glioblastoma		Grade 4	1 (8%)	4 (31%)	5 (20%)	

**Table 2:** Interhemispheric differences of Frontal Aslant Tract (FAT) variables reported according to tumor side for cases with bilaterally identifiable tract with spherical deconvolution tractography (n = 23). ADC—apparent diffusion coefficient; HMOA—hindrance modulated orientational anisotropy; SD—standard deviation.

Spherical Deconvolution FAT Variables	Contralateral Side (SD)	Tumor Side (SD)	Statistics
Mean Volume (mm^3^)	13.33 (4.46)	6.53 (4.72)	***p*** **< 0.001**
Mean Length (mm)	69.90 (4.07)	75.24 (13.06)	***p*** **= 0.043**
Mean HMOA	0.013 (0.002)	0.011 (0.002)	***p*** **= 0.001**
Mean ADC (×10^−3^ mm^2^/s)	0.682 (0.045)	0.770 (0.117)	***p*** **< 0.001**

**Table 3:** Analyses of association of verbal SMA syndrome development with mean interhemispheric volume asymmetry (VA) indexes as determined by dominance side and identified with spherical deconvolution tractography. SD—standard deviation. The cases affected by the verbal syndrome have a negative mean FAT-VA value, therefore asymmetry oriented towards the nondominant hemisphere, while the non-affected cases have a positive mean FAT-VA oriented towards the dominant hemisphere.

Interhemispheric Spherical Deconvolution Value	Verbal SMA Syndrome (N = 6)	No Verbal Syndrome (N = 17)	Statistics
Mean FAT-VA (SD)	−0.68 (0.904)	0.42 (0.794)	***p*** **= 0.010**

## Data Availability

The original clinical data presented in the study are included in the article and in the Supplementary Materials; further inquiries can be directed to the corresponding authors.

## Supplementary Materials

The following supporting information can be downloaded at: https://www.mdpi.com/article/10.3390/cancers16223739/s1, Figure S1: Box plot representations of mean values of spherical deconvolution interhemispheric FAT volume asymmetry estimated according to dominance, plotted for the laterality of the tumor in the dominant and nondominant hemispheres. Figure S2: Box plot representations of interhemispheric FAT volume asymmetry (VA) estimated according to dominance, plotted for the presence of tumor in the dominant and nondominant hemispheres and grouped according to onset of SMA syndrome or not after surgery. Table S1: General patient features, pathology, tumor location and extension, surgical protocol, and volumetric surgical outcomes in the cohort. Table S2: Postoperative functional outcomes and clinical course of supplementary syndrome in the studied cohort. Table S3: Functional SMA syndrome features recorded in n = 12 patients, subdivided in motor and verbal components. Table S4: Analysis of FAT features in the hemispheres affected by tumor and association with SMA syndrome onset. Table S5: Analysis of FAT features in the hemispheres contralateral to tumor side and association with SMA syndrome onset. Table S6: Analyses of possible association of SMA syndrome development with mean interhemispheric FAT volume asymmetry indexes as determined by dominance side, among n = 23 with bilaterally identifiable tract. Table S7: Analyses of possible association of motor SMA syndrome development with mean interhemispheric FAT volume asymmetry indexes (FAT-VA) as determined by dominance side, among n = 23 with bilaterally identifiable tract.
