# Supplementary material for: Asymmetry of the Frontal Aslant Tract and Development of Supplementary Motor Area Syndrome
Source: Cancers (Basel). 2024 Nov 5;16(22):3739. doi: 10.3390/cancers16223739 (PMC11592341; doi:10.3390/cancers16223739)
Supplement: Supplementary file 1 [file cancers-16-03739-s001.zip › cancers-3219451-supplementary.pdf]

Supplementary Materials: Asymmetry of the Frontal Aslant Tract and Development of Supplementary Motor Area Syndrome

Jahard M Aliaga-Arias, Josephine Jung, Jose Pedro Lavrador, Kapil Rajwani, Ana Mirallave-Pescador, Amy Jones, Hilary Wren, Richard Gullan, Ranj Bhangoo, Keyoumars Ashkan, Flavio Dell'Acqua and Francesco Vergani

Supplementary Materials

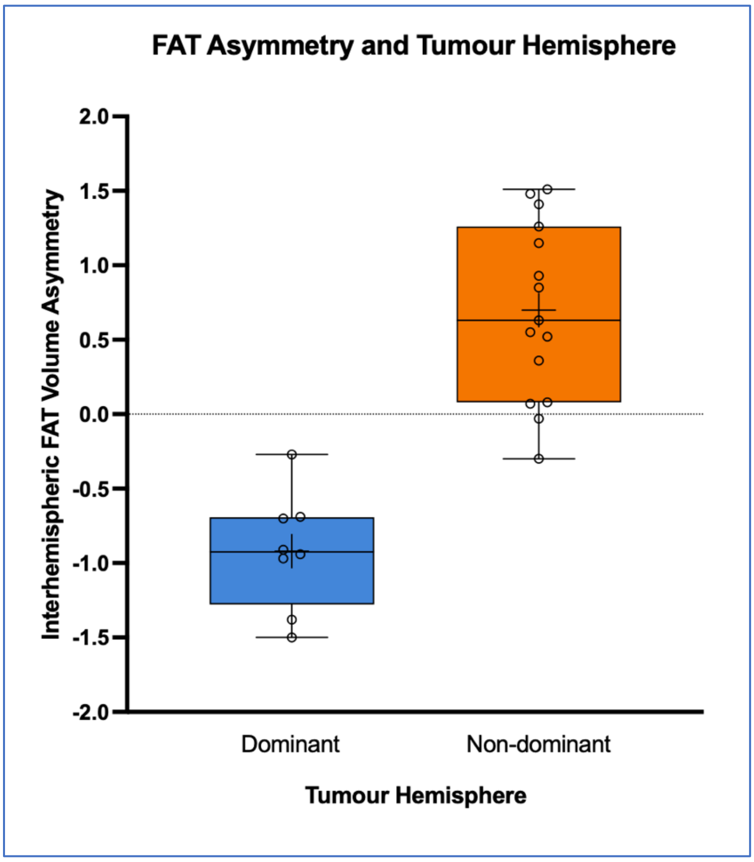

Figure S1. Box plot representations of mean values of spherical deconvolution interhemispheric FAT volume asymmetry (VA) estimated according to dominance, plotted for the laterality of the tumor in the dominant and nondominant hemisphere, demonstrating VA oriented concordantly contralaterally to the lesions with negative values for n=8 cases with tumors in the dominant side (mean -0.92) and positive values in n=15 with tumor in nondominant hemisphere (mean 0.70, $p<0.001$).

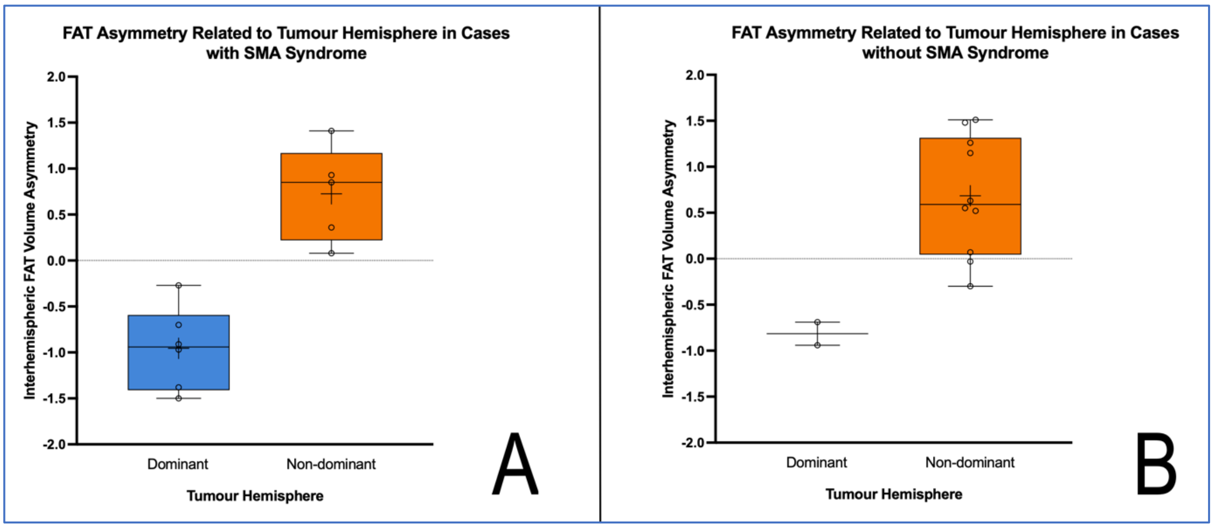

Figure S2. Box plot representations of interhemispheric FAT volume asymmetry (VA) estimated according to dominance, plotted for the present of tumor in the dominant and nondominant hemispheres and grouped according to (**A**) onset of SMA syndrome or (**B**) not after surgery. In both groups the VA remains oriented contralaterally to the lesions, with negative values for cases with tumors in the dominant side (means -0.95 in n=6 cases developing SMA syndrome and -0.81 in n=2 that did not develop the syndrome) and positive values in those with tumor in nondominant hemisphere (means 0.73 in n=5 cases developing SMA syndrome and 0.68 in n=10 not developing the syndrome, $p<0.001$ and $p=0.011$ respectively).

Table S1. General patient features, pathology, tumor location and extension, surgical protocol and volumetric surgical outcomes in the cohort (n=25).

Case N.	General Patient Features			Pathology				Tumor Location and Extension					Surgical	Volumetric Surgical Outcome	
	Age at Surgery	Gender	Handedness	Histology	IDH	1p/19q	WHO Grade	Tumor Side	SMA proper	preSMA	Cingulate	Preoperative Volume (cm³)	Protocol	Residue Volume (cm³)	EoR
**1**	36	Male	Right	Astrocytoma	mut		3	Nondominant	Yes	Yes	Yes	33.1	Asleep	0	100.00%
2	69	Male	Right	Oligodendroglioma	mut	Codel	2	Nondominant	Yes	Yes	No	3.6	Asleep	0	100.00%
3	30	Female	Right	Astrocytoma	mut		3	Dominant	No	Yes	Yes	106.1	Awake	7.2	93.21%
4	55	Male	Left	Oligodendroglioma	mut	Codel	3	Dominant	Yes	Yes	Yes	71.3	Awake	3.9	94.53%
5	37	Male	Right	Glioblastoma	wt		4	Nondominant	Yes	No	No	17.2	Asleep	0.6	96.51%
6	30	Male	Right	Astrocytoma	mut		3	Nondominant	Yes	Yes	Yes	113.9	Asleep	19.3	83.06%
7	53	Female	Right	Astrocytoma	mut		2	Nondominant	No	Yes	Yes	55.9	Awake	2.5	95.53%
8	42	Female	Right	Astrocytoma	mut		2	Dominant	Yes	Yes	Yes	123.3	Awake	86.7	29.68%
9	69	Male	Left	Glioblastoma	wt		4	Nondominant	No	Yes	Yes	44.2	Asleep	0	100.00%
10	48	Female	Right	Astrocytoma	mut		2	Nondominant	Yes	Yes	Yes	79.7	Awake	9.3	88.33%
11	32	Male	Right	Astrocytoma	mut		2	Dominant	Yes	Yes	No	39.5	Awake	0	100.00%
12	22	Female	Right	Oligodendroglioma	mut	Codel	2	Dominant	No	Yes	Yes	61	Awake	0	100.00%
13	49	Female	Right	Oligodendroglioma	mut	Codel	3	Dominant	Yes	Yes	No	15.3	Awake	0	100.00%
14	41	Female	Right	Oligodendroglioma	mut		3	Nondominant	No	Yes	No	9.8	Awake	0	100.00%
15	44	Female	Right	Astrocytoma	mut		4	Nondominant	Yes	Yes	Yes	46.1	Awake	2.3	95.01%
16	71	Female	Right	Glioblastoma	wt		4	Nondominant	Yes	No	No	9.4	Awake	0	100.00%

17	28	Female	Right	Glioblastoma	wt	4	Dominant	No	Yes	Yes	50.2	Awake	0	100.00%
18	56	Female	Right	Oligodendroglioma	mut Codel	3	Nondominant	Yes	Yes	Yes	107.2	Asleep	0	100.00%
19	31	Male	Right	Astrocytoma	mut	3	Nondominant	Yes	Yes	Yes	24.5	Awake	2.6	89.39%
20	29	Male	Right	Astrocytoma	mut	3	Nondominant	Yes	Yes	Yes	59.2	Awake	8.9	84.97%
21	45	Male	Right	Oligodendroglioma	mut Codel	3	Nondominant	Yes	Yes	Yes	46.8	Awake	3	93.59%
22	53	Male	Right	Astrocytoma	mut	3	Nondominant	No	Yes	Yes	195.9	Awake	12.5	93.62%
23	57	Female	Right	Glioblastoma	wt	4	Nondominant	Yes	No	Yes	12	Asleep	0	100.00%
24	51	Female	Right	Astrocytoma	mut	3	Nondominant	Yes	Yes	Yes	48.4	Awake	0.7	98.55%
25	24	Male	Right	Astrocytoma	mut	3	Dominant	Yes	Yes	Yes	43	Awake	5.5	87.21%

SMA= supplementary motor area; EoR= extent of resection.

Table S2. Postoperative functional outcomes and clinical course of supplementary syndrome in the studied cohort (n=25).

		Postoperative Functional Outcome									Clinical Outcome			
**Case N.**	**SMA Syndrome**	**MRC LUL**	**MRC LLL**	**MRC RUL**	**MRC RLL**	**Motor Initiation**	**Dexterity Deficit**	**Bimanual movement**	**Spontaneous Speech**	**SAS (Expressive/Receptive)**	**Syndrome Onset (POD)**	**Syndrome on POD 7**	**Discharge Destination**	**Postoperative LoS**
1	None	4	4	5	5	Intact	Yes	Intact	Intact	NA			Home	3
2	Motor	3	3	4	5	Impaired	Yes	Impaired	Intact	NA	1	Improved	Rehab	13
3	Motor and verbal	5	5	5	5	Impaired	No	NA	Mutism	NA	1	Improved	Home	9
4	Motor and verbal	4	1	5	5	Akinesia	Yes	Impaired	Impaired	NA	0	Improved	Rehab	7
5	None	5	5	5	3	Intact	No	NA	Intact	NA			Home	5
6	None	5	5	5	5	Intact	No	Intact	Intact	NA			Home	5
7	None	5	5	5	5	Intact	No	Intact	Intact	NA			Home	3
8	Verbal	5	5	5	5	Intact	NA	NA	Impaired	NA (NA/2)	1	Improved	Home	6
9	None	5	5	5	5	Intact	No	NA	Intact	NA			Home	6
10	Motor	1	2	5	5	Impaired	Yes	Impaired	Intact	NA	1	Improved	Rehab	14
11	Motor	5	5	5	5	Impaired	Yes	Impaired	Intact	20 (11/9)	0	Improved	Rehab	4

12	Verbal	5	5	5	5	Intact	No	No	Impaired	8 (2/6)	0	Improved	Home	5
13	Verbal	5	5	5	5	Intact	No	NA	Impaired	8 (3/5)	1	Resolved	Home	5
14	None	5	5	5	5	Intact	No	Intact	Intact	NA			Home	2
15	None	5	5	5	5	Intact	No	Intact	Intact	NA			Home	2
16	None	4	4	5	5	Intact	No	Intact	Intact	NA			Home	4
17	None	5	5	5	5	Intact	NA	NA	Intact	NA			Home	3
18	Verbal	5	4	5	4	Intact	NA	NA	Impaired	NA	1	Improved	Home	5
19	Motor	4	4	4	4	Impaired	Yes	Impaired	Intact	NA	0	Persistent	Home	3
20	None	5	5	5	5	Intact	No	Intact	Intact	NA			Home	3
21	None	5	5	5	5	Intact	No	Intact	Intact	NA			Home	5
22	Motor	4	4	5	5	Impaired	Yes	Impaired	Intact	NA	1	Resolved	Home	8
23	Motor	3	0	5	5	Akinesia	Yes	NA	Intact	NA	1	Improved	Home	9
24	None	5	5	5	5	Intact	Yes	NA	Intact	NA			Rehab	12
25	None	5	5	5	5	Intact	Yes	Intact	Intact	NA			Home	2

SMA= supplementary motor area; MRC= Medical Research Council motor power scale; LUL= left upper limb; LLL= left lower limb; RUL= right upper limb; RLL= right lower limb; SAS= Sheffield screening test aphasia score; POD= postoperative day; LoS= length of stay.

Table S3. Functional SMA syndrome features recorded in n=12 patients, subdivided in motor (n=8) and verbal (n=6) components.

SMA syndrome features		Cases (n=12)
**Motor (n=8)**	Motor initiation	8 (67%)
	Dyspraxia	7 (58%)
	Bimanual movement deficit	6 (50%)
	Strength	7 (58%)
	Akinesia/severe paresis	3 (25%)
**Verbal (n=6)**	Spontaneous speech	6 (50%)
	Mutism/severe dysphasia	2 (17%)

Table S4. Analysis of FAT features in the hemispheres affected by tumor and association with SMA syndrome onset (n=23) obtained with spherical deconvolution tractography data.

Tumor Side Spherical Deconvolution FAT Variables	SMA syndrome (n=11)	No syndrome (n=12)	Statistics
**Mean Volume (SD)**	5.86 (3.63)	7.13 (5.64)	t-test p=0.531
**Mean Length (SD)**	75.47 (10.86)	75.03 (15.30)	t-test p=0.938
**Mean HMOA (SD)**	0.011 (0.003)	0.011 (0.001)	t-test p=0.952
**Mean ADC (SD)**	0.790 x10^{-3}mm^2/s (0.150 x10^{-3}mm^2/s)	0.752 x10^{-3}mm^2/s (0.080 x10^{-3}mm^2/s)	t-test p=0-449

SD= standard deviation.

Table S5. Analysis of FAT features in the hemispheres contralateral to tumor side and association with SMA syndrome onset (n=25) from spherical deconvolution tractography data.

Contralateral Spherical Deconvolution FAT Variables	SMA syndrome (n=12)	No syndrome (n=13)	Statistics
**Mean Volume (SD)**	12.52 (3.59)	13.15 (5.48)	t-test p=0.741
**Mean Length (SD)**	68.99 (3.34)	70.88 (4.64)	t-test p=0.258
**Mean HMOA (SD)**	0.012 (0.002)	0.013 (0.002)	t-test p=0.196
**Mean ADC (SD)**	0.682 x10^{-3}mm^2/s (0.043 x10^{-3}mm^2/s)	0.673 x10^{-3}mm^2/s (0.040 x10^{-3}mm^2/s)	t-test p=0.320

SD= standard deviation.

Table S6. Analyses of possible association of SMA syndrome development with mean interhemispheric FAT volume asymmetry indexes (FAT-VA) as determined by dominance side, among n=23 with bilaterally identifiable tract with spherical deconvolution.

Interhemispheric Spherical Deconvolution Value	SMA syndrome (n=11)	No syndrome (n=12)	Statistics
**Mean FAT-VA (SD)**	-0.19 (0.991)	0.43 (0.828)	t-test p=0.115

SD= standard deviation.

Table S7. Analyses of possible association of motor SMA syndrome development with mean interhemispheric FAT volume asymmetry indexes (FAT-VA) as determined by dominance side, among n=23 with bilaterally identifiable tract with spherical deconvolution.

Interhemispheric Spherical Deconvolution Value	Motor SMA syndrome (n=7)	No motor syndrome (n=16)	Statistics
**Mean FAT-VA (SD)**	0.06 (1.033)	0.22 (0.924)	t-test p=0.526

SD= standard deviation.
